# Occurrence of Diarrheal Disease among Under-Five Children and Associated Sociodemographic and Household Environmental Factors: An Investigation Based on National Family Health Survey-4 in Rural India

**DOI:** 10.3390/children9050658

**Published:** 2022-05-03

**Authors:** Jay Saha, Sabbir Mondal, Pradip Chouhan, Mulazim Hussain, Juan Yang, Asma Bibi

**Affiliations:** 1 Department of Geography, University of Gour Banga (UGB), Malda 732103, WB, India; sabbirmondal143@gmail.com (S.M.); pradipchouhanmalda@gmail.com (P.C.); 2 The Children Hospital, Pakistan Institute of Medical Sciences (PIMS), Shaheed Zulfiqar Ali Bhutto Medical University (SZABMU), Islamabad 44000, Pakistan; drkhara786@gmail.com; 3 Chinese Academy of Science and Technology for Development, Beijing 100038, China; 4 Independent Researcher, Lahore 54000, Pakistan; asmatoorkham@gmail.com

**Keywords:** diarrheal disease, under-five children, environmental factors, NFHS, rural India

## Abstract

Diarrheal disease is a significant public health problem leading to mortality and morbidity among children aged 0–59 months in rural India. Therefore, the rationale of this study was to identify the sociodemographic and environmental predictors associated with diarrhea among under-five children in rural India. A total of 188,521 living children (0–59 months) were studied from the National Family Health Survey-4, (NFHS-4) 2015–2016. Bivariate and binary logistic regression models were carried out from the available NFHS-4 data for selected sociodemographic and environmental predictors to identify the relationship of occurrence of diarrhea using STATA 13.1. In rural India, children aged 12–23 months, 24–35 months, 36–47 months, and 48–59 months were significantly improbable to suffer diarrheal disease. Children of the female sex, as well as children of scheduled tribes (ST) and other backward classes (OBC), were less likely to experience diarrhea. The disease was more likely to occur among children of scheduled castes (SC); Muslim or other religions; children belonging to central, eastern, and western regions; children with low birth weight; as well as children with improper stool disposal and rudimentary roof materials. In the rural parts of India, sociodemographic and household environmental factors were most influential. Effective community education; improved handwashing practices; pure water supply; and proper waste disposal, including building and utilizing latrines, would help reduce the burden of diarrheal disease in children.

## 1. Introduction

Worldwide, diarrheal disease is a significant population health hazard. It is a leading cause of mortality and morbidity among under-five children in developing countries. According to the WHO, abnormal fecal discharge characterized by frequent and fluid stool usually results from a non-invasive infection of the small intestine. It involves fluids and electrolytes loss without blood and pus [1]. A wide range of microbial pathogens, such as bacteria, viruses, and parasites, can infect the gastrointestinal tract. Such pathogens can be acquired from fecally contaminated food, water, fingers, etc., through the fecal–oral route [1,2]. The leading causes of diarrhea deaths are severe dehydration and fluid loss for most people and children [1]. Diarrhea is treated with an ORS, i.e., a solution of clean water, sugar, and salt [3].

A proportion of 3 out of 1000 children under five years died due to diarrhea in 2016, accounting for approximately 8 percent of all deaths among children under age five worldwide in 2017 [4]. Globally, there are nearly 1.7 billion cases of childhood diarrheal disease every year, killing around 525,000 under-five children [1]. Most deaths from diarrhea develop among children less than two years of age living in South Asia and sub-Saharan Africa [5]. From 2000 to 2017, the total annual number of diarrhea-related deaths among children under five decreased by 60 percent [6]. According to the NFHS-4 report, 9.19% of under-five children in India (8.24% in urban and 9.57% in rural areas) had diarrhea within two weeks before the survey [7]. Children in rural India are often treated wrongly for diarrhea and pneumonia, the two leading killers of children [8]. Many cases of diarrheal disease are not diagnosed, either because they are mild and self-limiting or the patient does not seek medical attention [7].

Pneumonia and diarrhea account for 29% of all child deaths globally [9] and 50% of all child deaths in India [10]. The Integrated Global Action Plan for the Prevention and Control of Pneumonia and Diarrhea (GAPPD) was launched by the WHO/UNICEF to reduce these two preventable diseases, i.e., diarrhea and pneumonia [9]. The WHO/ UNICEF report, ‘Diarrhea: Why children are still dying and what can be done’, lays out a ‘seven-point plan’ that includes a treatment package to reduce childhood diarrhea deaths [11,12]. To reduce diarrheal disease deaths in India, the union health ministry, in association with a few international health organizations, launched the ‘Intensified Diarrhea Control Fortnight (IDCF)-2015 program’ [13]. Accredited social health activist (ASHA) workers will carry out various activities in the remote and distant parts of the states to distribute ORS packets to every family with children under five, in addition to group counseling [14]. The Integrated Child Development Services (ICDS) scheme is an extensive program launched by the Government of India on 2 October 1975 to encourage maternal and child health and nutrition not only in India to reduce childhood malnutrition and associated morbidities among under-five children [15].

Several child sociodemographic, nutritional, health, and environmental predictors are directly and indirectly associated with diarrheal disease, including child sex, child age, maternal education, maternal age, household income [16,17,18,19,20,21,22,23,24,25,26,27,28], breastfeeding status, nutritional status [17,20,23,26], drinking water quality, sanitation condition, stool disposal site, and household dwelling characteristics [16,23,24,26,27,29,30,31].

Although there is a higher level of diarrhea disease in rural India than in urban India, few studies have been conducted at the district level to assess diarrheal illness and its associated factors. There have also been no studies at the country level specifically addressing the rural parts of the country, using a nationally representative survey to show associated factors of diarrhea. Therefore, evidence-based information is needed for a children’s health development strategy to prevent and reduce the severity of diarrheal disease in under-five children in rural India. Thus, with this study, we intend to fill this gap by achieving the aim of identifying the sociodemographic and environmental factors associated with the occurrence of diarrhea among children under five years of age in rural India.

## 2. Data and Methods

### 2.1. Study Design and Period

A cross-sectional study has been conducted using the fourth round of the NFHS. Data were collected between 20 January 2015 and 4 December 2016.

### 2.2. Data Source and Study Sample

For this study, data from the fourth round of the NFHS (2015–16) were used, which were nationally representative. The purpose of this survey was to gather essential information on family planning, fertility, maternal and child health, under-five nutrition, anemia, infants, maternal–child mortality indicators, other adult health issues, HIV/AIDS-related knowledge, attitudes, behavior, and domestic violence [7].

In total, 188,521 living children (0–59 months) from rural India of different sociodemographic and household environmental characteristics were studied from the NFHS-4. The data used in this study were retrieved from the public domain after describing the study’s objective.

### 2.3. Sampling Design and Technique

The NFHS-4 samples were collected using a stratified two-stage sampling design. In the first stage of sample selection, 28,586 primary sampling units (PSUs) were selected, 130 PSUs were selected from the slums listed by municipal corporation offices (MCOs) and the rest were selected from the census sampling frame (8397 in urban areas and 20,059 in rural areas) [7]. The rural sample was selected through a two-stage sample design with villages as the PSUs (chosen with probability proportional to size [PPS]) and followed by a random selection of 22 households in each PSU in rural areas in the second stage after conducting a complete mapping and household listing operation in the selected first-stage units [7].

### 2.4. Outcome Variable

‘Had diarrhea recently’ is the outcome variable of this study. It is defined as having three or more loose or watery stools in 24 h within the past two weeks preceding the data collection, as reported by the mother/caretaker of the child. This variable was coded as ‘no’ diarrhea (coded as ‘0’) and ‘yes’ diarrhea (assigned code ‘1’) for the smooth running of the bivariate and multivariate logistic regression model.

### 2.5. Predictor Variables

#### 2.5.1. Sociodemographic Predictors

For the analysis, various sociodemographic and household environmental predictors were included in this study, identified from the pre-existing literature, and associated with diarrheal disease among under-five children. Children’s sociodemographic predictor variables included various entities: social group (scheduled caste (SC), scheduled tribe (ST), other backward classes (OBCs), and other), religion (Hindu, Muslim, and other), current age of the mother (classified into three groups: 15–24, 25–34, and 35–49 years), education of mother/caregivers (illiterate, primary and secondary, and above), wealth index (poor, middle, and rich), body mass index (BMI) of mother (underweight: <18.5 kg/m^2^; normal weight: 18.5–24.9 kg/m^2^; and overweight and obesity: >25 kg/m^2^), region (north, central, east, northeast, west, and south), child’s sex (male and female), current age of child in months (0–11, 12–23, 24–35, 36–47, and 48–59 months), birth order number (1, 2–3, and 4 and more), birth weight (normal birth weight: ≥2500 gm; low birth weight: <2500 gm), nutritional status (the WHO Multicentre Growth Reference Study weight-for-age of under-five children was computed; nutritional status is classified as: normal (above minus two standard deviations (−2 SD)) or undernourished (below minus two standard deviations (−2 SD) and minus three standard deviations (−3 SD) from the median of the reference population)), and currently breastfeeding (no or yes).

#### 2.5.2. Household Environmental Predictors

Children’s environmental predictor variables included sources of drinking water (unimproved vs. improved (including piped water, public taps, standpipes, tube wells, boreholes, protected dug wells and springs, rainwater, and community reverses osmosis (RO) plants [7])), toilet facilities (unimproved and improved (including any non-shared toilet of the following types: flush/pour flush toilets to piped sewer systems, septic tanks, and pit latrines; ventilated improved pit (VIP)/biogas latrines; pit latrines with slabs; and twin pit/composting toilets [7]]), toilet facility shared with other households (not shared and shared), disposal of youngest child’s stool when not using the toilet (proper disposal, i.e., adequately disposed of, whether a child used a toilet or latrine, put/rinsed into a toilet, or it was buried [7]) and improper disposal), roof material (concrete and rudimentary roof), and floor material (clean and unclean).

### 2.6. Statistical Analyses

All analyses were completed using the statistical package in data science software STATA version 13.1 (StataCorp LP, College Station, TX, USA). In the preliminary analysis section, inferential data analysis and general descriptive analysis were carried out to explain the weighted percentage of the sample of children and the number of living children (total sample, *n*). The sample survey data were weighted to represent the under-five children’s structure of rural India, using the weighting factors provided by the NFHS. Differentials, bivariate association, and Pearson’s chi-square test analyses were used to explain the occurrence of diarrhea. A multivariate binary logistic regression model was used to identify the sociodemographic and environmental predictors of diarrhea occurrence among children aged 0–59 months. The power of the associations between predictors and the outcome variable in the analysis depended on crude odds ratios (CORs) and adjusted odds ratios (AORs) with 95% confidence intervals and the level of significance at *p* value < 0.05.

## 3. Results

### 3.1. Sociodemographic Characteristics of Living Under-Five Children

A total of 188,521 living children aged 0–59 months in rural India were included in this study. The majority of children’s (81.49%) religion was Hindu, and nearly one-fourth of the children (23.77%) belonged to a scheduled caste. The majority of mothers (54.73%) were in the age group of 25–34 years. Half of the mothers/caregivers (50.27%) had no education to the primary level. Nearly one-fourth (28.22%) of were underweight. Of the total, 29.63% of children were from the east, and 28.88% were from central regions. About half of the mothers (47.49%) were second or third in birth order, and more than half of the children’s families (59.89%) had poor wealth conditions. Among all living under-five children, 51.88% were male, 38.25% were undernourished, 17.88% had low birth weight, and 68.48% had a history of breastfeeding (Table 1).

### 3.2. Household Environmental Characteristics of Living Under-Five Children

Concerning environmental predictors, about one-tenth of children (10.24%) used drinking water from unimproved sources, about 37.19% of children had improved toilet facilities, 17.16% of children had toilet facilities shared with other households, and about one-third of children (75.89%) disposed of their stool improperly. Furthermore, of the total children, 56.55% had a dwelling with an unclean floor, and 17% of children had a rudimentary roof in rural India (Table 2).

### 3.3. Occurrence of Diarrhea according to Different Sociodemographic Predictors

The total occurrence of diarrhea among under-five children was 9.57% in rural India. The result in Table 3 shows that the development of diarrhea was higher among the children whose mothers/caregivers had no education to the primary level of education. The occurrence of diarrhea among children belonging to SC was 9.89% and 10.32% among children belonging to the Muslim religion. The prevalence of diarrhea was higher among children with mothers aged 15–24 years (10.9%) and with an underweight body mass index (10.19%). The occurrence of diarrhea was high among the children from the central region (14.3%) and those from households categorized as poor (9.97%) according to the wealth index. The occurrence of diarrhea was the highest among children aged 0–11 months (14.41%) and 12–23 months (13.89%), male children (9.88%), currently breastfeeding children (10.55%), children with a birth order of three or more (10.6%), children who had a low birth weight (10.76%), and children with undernourished nutritional status (10.37%).

### 3.4. Occurrence of Diarrhea according to Different Household Environmental Characteristics

In rural India, diarrheal diseases were also remarkably higher among those children whose households have unimproved toilet facilities (10.06%) compared to those households with improved toilet facilities (8.84%) (*p* ≤ 0.001). Diarrhea was also significantly higher among those children who had shared (10.1%) their toilet facility with other households than those who did not share toilet facilities (8.36%) (*p* ≤ 0.001). Surprisingly, diarrhea was higher among children whose families had used improved sources (9.69%) of drinking water than those children who had used unimproved sources (7.74%) (*p* ≤ 0.001). The occurrence of diarrhea was significantly higher among children living in a dwelling with a dirty floor (10.29%) (*p* ≤ 0.001) compared to children who were living in a dwelling with a clean floor material (8.46%). Diarrhea occurrence was also higher among children living in a dwelling with rudimentary roof materials (10.4%) compared to those with concrete roofs (9.21%) (*p* ≤ 0.001) (Table 4).

### 3.5. Sociodemographic Predictors Associated with Diarrhea among Under-Five Children

A multivariate logistic regression model was used to evaluate the relative effect of the predictor variable on the outcome variable (Table 5). Childhood diarrhea was 0.811 and 0.902 times less likely to occur among children belonging to ST (AOR: 0.811, 95% CI (0.755, 0.872)) and OBCs (AOR: 0.902, 95% CI (0.851, 0.956)), respectively, compared to SC children. Diarrheal disease was also 1.217 and 1.163 times more likely to occur among Muslim children (AOR: 1.217, 95% CI (1.128, 1.313)) and other religious groups (AOR: 1.163, 95% CI (1.062, 1.272), respectively, than Hindu children and more likely to develop in children from the central region (AOR: 1.510, 95% CI (1.410, 1.617)), east region (AOR: 1.077, 95% CI (1.002, 1.157)), and west region (AOR: 1.201, 95% CI (1.095, 1.317)). In both crude and adjusted analyses, the educational level of mothers/caregivers had no statistically significant association with childhood diarrhea.

In the crude analysis, diarrheal morbidity was 0.785 and 0.712 times less likely to develop among the children of mothers aged 25–34 years (COR: 0.785, 95% CI (0.759, 0.811)) and 35–49 years (COR: 0.712, 95% CI (0.671, 0.755)), respectively, compared to the children of younger mothers (15–24 years). Children from households categorized as rich according to the wealth index were significantly less likely to have diarrheal disease than children from poor households (COR: 0.942, 95% CI (0.904, 0.982)). Diarrhea was significantly less likely to develop among children of mothers with normal BMI (COR: 0.934, 95% CI (0.901, 0.968)) and children of overweight obese mothers (COR: 0.860, 95% CI (0.812, 0.912)).

Diarrhea was less likely to develop among children aged 12–23 months (AOR: 0.928, 95% CI (0.876, 0.983)), 24–35 months (AOR: 0.579, 95% CI (0.54, 0.617)), 36–47 months (AOR: 0.394, 95% CI (0.367, 0.424)), and 49–59 months (AOR: 0.313, 95% CI (0.289, 0.339)) compared to children aged 0–11 months. Diarrheal disease was 0.897 times less likely to prevail among female children (AOR: 0.897, 95% CI (0.859, 0.937)). The odds of emergent diarrhea were 1.135 times higher among children with low birth weight (AOR: 1.135, 95% CI (1.074, 1.201)) than those with normal birth weight. The odds of developing diarrhea were 1.097 times higher among undernourished children (AOR: 1.097, 95% CI (1.047, 1.149)) than well-nourished children. Diarrhea occurred more among children whose mothers had higher birth order (4+) (COR: 1.086, 95% CI (1.038, 1.136)) compared to first-birth-order children and was more likely to occur among those children who were currently breastfeeding (COR: 1.409, 95% CI (1.360, 1.461)) (Table 5).

### 3.6. Household Environmental Predictors Associated with Diarrhea among Under-Five Children

A crude analysis also found that the odds of developing diarrhea were 1.135 times higher among children whose families used unimproved toilet facilities (COR: 1.135, 95% CI (1.097, 1.174)) than children whose families used improved toilet facilities. The odds of developing diarrhea were 1.288 times higher among children of families who shared toilet facilities with other households (COR: 1.288, 95% CI (1.210, 1.371)) than children of families who did not share toilet facilities with others. Children living in households with unclean floor materials were 1.139 times more likely to have suffered diarrheal disease (COR: 1.139, 95% CI (1.102, 1.177)) than those children from houses with clean floor materials (Table 5).

Surprisingly, the emergence of diarrhea was 1.115 times more likely among children whose families used improved sources of drinking water (AOR: 1.115, 95% CI (1.038, 1.197)) compared to children of families who used unimproved water sources. The occurrence of diarrhea was 1.061 times more likely among children whose families improperly disposed of children’s stool (AOR: 1.061, 95% CI (1.002, 1.124)) compared to those children whose families properly disposed of stool. Children living in households with rudimentary roof materials were 1.113 times more likely (AOR: 1.113, 95% CI (1.048, 1.182)) to have suffered diarrheal disease than those children from houses with concrete roof materials (Table 5).

## 4. Discussion

In the present study, we investigated multiple sociodemographic and household environmental factors linked to diarrhea among under-five children. Various factors determine the prevalence of this disease in India. The occurrence of diarrhea has been reduced in India, but this disease is predominantly high in rural areas of India. According to the NFHS-4, about 9% of under-five children were adversely affected by diarrhea-related conditions.

This study depicts that social groups/castes are significantly associated with diarrhea disease. Children belonging to the STs, OBCs, and others were associated with a lower risk of diarrheal illness than SC children. A similar finding was reported in other studies conducted in India [32]. The present study also shows that religion is another determining factor. Children belonging to the Muslim religion were 21% more at risk of developing diarrhea disease than Hindu children. This result is consistent with previous studies conducted in India [27,32]. Recent research also showed that higher risk of diarrhea among ST and Muslim children might be due to deprivation of safe drinking water and toilet facilities. A study conducted in India showed similar findings, i.e., diarrhea was found to occur more among ST and Muslim children due to a lack of safe water, sanitation, and hygiene at home [32,33].

Multivariate statistics were also used to analyze maternal factors, such as maternal age and education, although no significant relationships were observed. On the other hand, a crude analysis of these variables showed a significant association. Women with secondary or higher education levels were less likely to have children with this disease than illiterate or mothers with a primary level of education. Similar findings were reported in Ethiopia [25], India [33], and Bangladesh [20].

A crude analysis of the wealth index indicated that wealthy and middle-income families had a lower risk of diarrhea disease than low-income families, who had less adequate access to safe drinking water and sanitation facilities. The findings of the current study revealed that diarrhea was reduced by 6% in rural children from rich households according to the wealth index compared to those from poor households. Similar results were observed in previous studies in India [27,33]. The household wealth index was determined based on a range of consumer items, sources of drinking water, and sanitation facilities, including dwelling characteristics, so the wealth index variable was excluded from the multivariate analysis. Different characteristics are included in the multivariate analysis of household environmental factors.

The study also revealed that children of underweight mothers had a 9% higher disease than children of normal-weight mothers. Recent studies and scientific knowledge also showed that undernourished mothers gave birth to malnourished and low-immune-system children susceptible to diseases such as diarrhea. The increased vulnerability to infections may, in part, be caused by injury of immune function by undernutrition [34]. The geographical region of the resident is considered an essential factor determining the risk of disease because the geographical area also determines the unequal availability of safe drinking water and sanitation. The study results also indicate that children from the central regions were 1.5 times more affected by diarrhea than those from southern and western regions. However, under-five children from the rural northeast region were 28% less likely to be affected by this disease than those from the rural northern part of India [34].

The study also revealed that female children had a 10% lower risk of diarrhea than the male children. This result is in agreement with studies in Bangladesh [20] and India [35], both of which reported a higher risk of diarrhea among male children than female children. There may be a sex-based differentiation in the pathophysiology of acute pediatric diarrhea that we do not yet comprehend. In this study, we found that the risk of diarrhea decreased by 40–69% in young children (aged 24 to 59 months) compared to infants (children aged 0–11 months). The risk of diarrhea was significantly higher among children aged 12–23 months than in older age groups. This result is also corroborated by a study in India [36] in which the lower-age-group or infant children were most affected by this disease because they had a weaker immune system. The 2015–2016 NFHS also reported that diarrhea occurrence remains high (7%) among the 0–11 month group, which is the period during which children start walking and are at increased risk of contamination from the environment [7].

The study also revealed that the children of women with a higher birth order were more likely to have diarrhea disease than the children of women with a lower birth order. In the current study, we also focused on birth weight factors, which revealed that lower-birth-weight children had a higher risk of diarrhea than normal-birth-weight children. This result is consistent with a rural community-based study in south India [37] in which malnourished and currently breastfeeding children were more likely to be affected by diarrhea disease. This finding is also corroborated by studies in West Bengal [38] and Pakistan [39,40].

The present study also revealed that environmental factors, such as drinking water sources, toilet facilities, child stool disposal, roof material, and floor material, were associated with diarrheal disease among under-five children. A previous study in Ethiopia represented a similar finding [16]. Improved toilet facilities were found to reduce the risk of diarrhea disease. The study authors stated that unimproved sanitation facilities increased the risk of diarrhea by 1% among children compared to improved facilities. Improved sanitation facilities significantly decrease morbidity and infections among children; this result is corroborated by a study in Pakistan [39,40]. Improper child stool disposal was associated with a 6% increased likelihood of diarrhea among children. Children living in homes with concrete roof materials had an 11% lower risk of diarrhea compared to children living in households with rudimentary roof materials. Another important finding was that the children living in dirty or unclean floor conditions had a 2% higher likelihood of diarrhea disease. This occurs because improper hygiene practices in children significantly increase infections [39], as children come in contact with the dirty floor, and pathogens are transmitted to children. This result is consistent with a study in Ethiopia [25]. The current study identified different sociodemographic and household environmental characteristics. Reducing associated risk factors and prevention practices in under-five children is crucial to controlling diarrhea-related morbidity and mortality.

### Limitations and Strengths of Study

This study did not include all modifiable linked factors, such as rotavirus, hand washing, behavioral characteristics, and others. It is important to discuss the limitations of this study. The results did not reveal a cause–effect association because in this study, we used cross-sectional data. More research is needed on the occurrence of diarrheal diseases among under-five children. The information used for analysis is self-reported, so the sampled data are susceptible to recall bias. Beyond the mentioned limitations, this study provides widespread evidence on the socioeconomic, demographic, and environmental factors associated with diarrheal disease among children based on a large-scale survey in India. In this study, we used a large sample with countrywide representation. This study is significant for public health intervention regarding the reduction of the problem of diarrheal disease among under-five children. Further study is recommended to investigate the factors associated with diarrhea using primary data, including all modifiable associated factors.

## 5. Conclusions

With this study, we found that different sociodemographic factors, such as caste, religion, economic condition, and women’s education, are significantly associated with diarrheal disease. Children belonging to scheduled castes, the Muslim religion, and economically backward families suffer the most from this disease. Household environmental factors, such as sources of drinking water, toilet facilities, child stool disposal, and household roof and floor materials, are also risk factors for diarrheal disease. Different sociodemographic and environmental factors should therefore be taken seriously. A targeted approach should be initiated to alleviate the burden of diarrheal disease by providing sufficient health care to socioeconomically disadvantaged women and children. The authors of the current study recommend that stakeholders and policy makers must address unfavorable environmental conditions by providing sanitation and cleanliness facilities. The government and non-governmental organizations (NGOs) might focus on improved drinking water sources and sanitation facilities, which reduce vulnerability to disease.

## Figures and Tables

**Table 1:** Sociodemographic characteristics of living children aged 0–59 months in rural India (from the NFHS-4, 2015–16).

Sociodemographic Characteristics	Frequency (*n*)	Percentage (%)
**Social group**		
SC	36,619	23.77
ST	42,684	13.37
OBC	71,685	45.91
Other	28,941	16.95
**Religion**		
Hindu	139,770	81.49
Muslim	25,532	13.88
Other	23,219	4.63
**Current age of mother in years**		
15–24	63,908	36.75
25–34	105,668	54.73
35–49	18,945	8.52
**Education of mother/caregiver**		
Illiterate and primary	95,541	50.27
Secondary and above	92,980	49.73
**Wealth index**		
Poor	113,792	59.89
Middle	38,361	20.68
Rich	36,368	19.43
**Maternal BMI**		
Underweight	48,629	28.22
Normal	118,211	61.20
Overweight and obesity	19,634	10.59
**Region**		
North	34,556	12.19
Central	54,593	28.88
East	43,904	29.63
Northeast	28,213	4.21
West	11,663	10.00
South	15,592	14.84
**Sex of child**		
Male	97,625	51.88
Female	90,896	48.12
**Current age of child in months**		
0–11	37,207	19.70
12–23	37,489	20.02
24–35	37,326	19.77
36–47	39,028	20.67
48–59	37,471	19.84
**Birth order number**		
1	67,038	36.46
2–3	88,217	47.49
3+	33,266	16.06
**Birth weight**		
Normal (≥2500 gm)	112,591	82.12
Low birth weight (<2500 gm)	23,423	17.88
**Nutritional status**		
Normal	109,124	61.75
Undernourished	62,395	38.25
**Currently breastfeeding**		
No	59,215	31.52
Yes	129,306	68.48

**Table 2:** Household environmental characteristics of living children aged 0–59 months in rural India (from the NFHS-4, 2015–16).

Environmental Characteristics	Frequency (*n*)	Percentage (%)
**Sources of drinking water**	23,844	10.24
Unimproved	154,383	89.76
Improved		
**Toilet facilities**		
Unimproved	96,059	62.81
Improved	71,075	37.19
**Toilet facility shared with other h. h.**		
Not shared	69,191	82.84
Shared	13,290	17.16
**Disposal of youngest child’s stool**		
Proper disposal	49,544	24.11
Improper disposal	136,140	75.89
**Main roof material**		
Concrete roof	142,676	83.00
Rudimentary roof	28,601	17.00
**Main floor material**		
Clean	78,205	43.45
Unclean	100,212	56.55

**Table 3:** Occurrence of diarrheal diseases according to sociodemographic characteristics of respondents in rural India (from the NFHS-4, 2015-16).

Sociodemographic Characteristics	Had Diarrhea Recently		Pearson’s χ^2^ Value	*p*-Value
	No (%)	Yes (%)		
**Social group**			232.06	<0.001
SC	90.11	9.89		
ST	91.67	8.33		
OBC	90.12	9.88		
Other	90.26	9.74		
**Religion**			223.84	<0.001
Hindu	90.43	9.57		
Muslim	89.68	10.32		
Other	92.84	7.16		
**Current age of mother in years**			256.40	<0.001
15–24	89.10	10.90		
25–34	91.19	8.81		
35–49	91.35	8.65		
**Education of mother/caregiver**			0.1122	0.738
Illiterate and primary	90.27	9.73		
Secondary and above	90.6	9.40		
**Wealth index**			7.90	0.01
Poor	90.03	9.97		
Middle	90.56	9.44		
Rich	91.55	8.45		
**Maternal BMI**			28.39	<0.001
Underweight	89.81	10.19		
Normal	90.55	9.45		
Overweight and obesity	91.36	8.64		
**Region**			1.30 × 10^3^	<0.001
North	92.33	7.67		
Central	86.82	13.18		
East	90.85	9.15		
Northeast	95.96	4.04		
West	90.47	9.53		
South	93.47	6.53		
**Sex of child**			31.70	<0.001
Male	90.12	9.88		
Female	90.77	9.23		
**Current age of child in months**			3.00 × 10^3^	<0.001
0–11	85.59	14.41		
12–23	86.11	13.89		
24–35	91.08	8.92		
36–47	94.01	5.99		
48–59	95.25	4.75		
**Birth order number**			12.85	0.002
1	90.80	9.20		
2–3	90.50	9.50		
3+	89.40	10.6		
**Birth weight**			78.32	<0.001
Normal (≥2500 gm)	90.96	9.04		
Low birth weight (<2500 gm)	89.24	10.76		
**Nutritional status**			42.29	<0.001
Normal	90.74	9.26		
Undernourished	89.63	10.37		
**Currently breastfeeding**			355.03	<0.001
No	92.58	7.42		
Yes	89.45	10.55		

**Note:** NFHS-4, rural India, 2015–16. Percentages are computed by applying sample weight.

**Table 4:** Occurrence of diarrheal diseases by household environmental characteristics of respondents in rural India (from the NFHS-4, 2015–16).

Environmental Characteristics	Had Diarrhea Recently		Pearson’s χ^2^ Value	*p*-Value
	No (%)	Yes (%)		
**Sources of drinking water**			82.08	<0.001
Unimproved	92.26	7.74		
Improved	90.31	9.69		
**Toilet facilities**			54.04	<0.001
Unimproved	89.94	10.06		
Improved	91.16	8.84		
**Toilet facility shared with other HH.**			63.24	<0.001
Not shared	91.64	8.36		
Shared	89.90	10.10		
**Disposal of youngest child’s stool**			91.32	<0.001
Proper	92.30	7.70		
Improper	89.85	10.15		
**Main roof material**			35.74	<0.001
Concrete roof	90.79	9.21		
Rudimentary roof	89.43	10.57		
**Main floor material**			60.54	<0.001
Clean	91.54	8.46		
Unclean	89.71	10.29		
**Rural India**	90.43	9.57		

**Table 5:** Multivariable binary logistic regression analysis of sociodemographic and environmental factors associated with diarrhea among children aged 0–59 months in rural India, (from the NFHS-4, 2015–16).

Variables	Crude OR	95% CI	Adjusted OR	95% CI
**Social group** (SC †)	1.00		1.00	
ST	0.727 ***	(0.691–0.764)	0.811 ***	(0.755–0.872)
OBC	0.989	(0.948–1.031)	0.902 ***	(0.851–0.956)
Other	0.997	(0.947–1.050)	0.992	(0.922–1.067)
**Religion** (Hindu †)	1.00		1.00	
Muslim	1.122 ***	(1.074–1.173)	1.217 ***	(1.128–1.313)
Other	0.694 ***	(0.657–0.732)	1.163 ***	(1.062–1.272)
**Current age of mother in years** (15–24 †)	1.00		-	
25–34	0.785 ***	(0.759–0.811)	-	-
35–49	0.712 ***	(0.671–0.755	-	-
**Education of mother/caregiver** (Illiterate and primary †)	1.00		1.00	
Secondary and above	1.005	(0.974–1.037)	1.025	(0.975–1.077)
**Wealth index** (Poor †)	1.00		-	
Middle	0.984	(0.946–1.024)	-	-
Rich	0.942 ***	(0.904–0.982)	-	-
**Maternal BMI** (Underweight †)	1.00		-	
Normal	0.934 ***	(0.901–0.968)	-	
Overweight and obesity	0.860 ***	(0.812–0.912)	-	-
**Region** (North †)	1.00		1.00	
Central	1.454 ***	(1.390–1.520)	1.510 ***	(1.410–1.617)
East	0.987	(0.939–1.037)	1.077 **	(1.002–1.157)
Northeast	0.601 ***	(0.564–0.640)	0.719 ***	(0.647–0.800)
West	1.041	(0.968–1.120)	1.201 ***	(1.095–1.317)
South	0.696 ***	(0.646–0.750)	0.788 ***	(0.718–0.864)
**Sex of child** (Male †)	1		1.00	
Female	0.914 ***	(0.886–0.943)	0.897 ***	(0.859–0.937)
**Current age of child in months** (0–11 †)	1.00		1.00	
12–23	0.968	(0.928–1.009)	0.928 **	(0.876–0.983)
24–35	0.602 ***	(0.575–0.631)	0.579 ***	(0.543–0.617)
36–47	0.393 ***	(0.373–0.414)	0.394 ***	(0.367–0.424)
48–59	0.318 ***	(0.300–0.336)	0.313 ***	(0.289–0.339)
**Birth order number** (1 †)	1.00		1.00	
2–3	1.032 *	(0.997–1.069)	1.013	(0.966–1.063)
3+	1.086 ***	(1.038–1.136)	1.039	(0.967–1.115)
**Birth weight** (Normal †)	1.00		1.00	
Low birth weight	1.231 ***	(1.176–1.289)	1.135 ***	(1.074–1.201)
**Nutritional status** (Normal †)	1.00		1.00	
Undernourished	1.117 ***	(1.081-1.155)	1.097 ***	(1.047–1.149)
**Currently breastfeeding** (No †)	1.00		-	
Yes	1.409 ***	(1.360–1.461)	-	-
**Sources of drinking water** (Unimproved †)	1.00		1.00	
Improved	1.266 ***	(1.203–1.332)	1.115 ***	(1.038–1.197)
**Toilet facilities** (Improved †)	1.00		1.00	
Unimproved	1.135 ***	(1.097–1.174)	1.017	(0.962–1.075)
**Toilet facility shared with other h. h.** (Not shared †)	1.00		-	
Shared	1.288 ***	(1.210–1.371)	-	-
**Disposal of youngest child’s stools** (Proper †)	1.00		1.00	
Improper	1.197 ***	(1.154–1.242)	1.061 **	(1.002–1.124)
**Main roof material** (Concrete roof †)	1.00		1.00	
Rudimentary roof	1.140 ***	(1.092–1.189)	1.113 ***	(1.048–1.182)
**Main floor material** (Clean †)	1.00		1.00	
Unclean	1.139 ***	(1.102–1.177)	1.026	(0.974–1.081)
**Constant**			0.124 ***	(0.110–0.141)
**Pseudo R2**			0.0367	
**Log-likelihood**			−29,756.176	
**Probability χ^2^**			0.0000	

OR = odds ratio, SC = scheduled caste, ST = scheduled tribe, OBC= other backward class, h. h. = households, CI = confidence interval in parentheses, *** *p* < 0.01, ** *p* < 0.05, * *p* < 0.1, † = reference category.

## Data Availability

The general datasets are available from the Demographic Health Surveys (DHS) repository. Specifically, the data used for this study are available from the corresponding author upon reasonable request.

## References

[1] WHO Diarrhoeal disease. https://www.who.int/news-room/fact-sheets/detail/diarrhoeal-disease.

[2] Farthing M.J.G., Kelly P. (2007). Infectious diarrhoea. Medicine.

[3] NHP ORS Day. https://www.nhp.gov.in.

[4] UNICEF Diarrhoea—UNICEF DATA. https://data.unicef.org/topic/child-health/diarrhoeal-disease/.

[5] UNICEF (2015). WHO Levels & Trends in Child Mortality: Report 2015.

[6] UNICEF UNICEF Data: Monitoring the Situation of Children and Women. https://data.unicef.org/resources/countdown-2030-tracking-progress-towards-universal-coverage-womens-childrens-adolescents-health/.

[7] International Institute for Population Sciences (IIPS); ICF National Family Health Survey—4: 2015-16. http://rchiips.org/nfhs/.

[8] Mohanan M., Vera-Hernández M., Das V., Giardili S., Goldhaber-Fiebert J.D., Rabin T.L., Raj S.S., Schwartz J.I., Seth A. (2015). The know-do gap in quality of health care for childhood diarrhea and pneumonia in rural India. JAMA Pediatr..

[9] WHO, UNICEF (2013). Ending Preventable Child Deaths from Pneumonia and Diarrhoea by 2025: The Integrated Global Action Plan for Pneumonia and Diarrhoea (GAPPD). https://www.who.int/publications-detail-redirect/the-integrated-global-action-plan-for-prevention-and-control-of-pneumonia-and-diarrhoea-(gappd).

[10] The Million Death Study Collaborators (2010). Causes of neonatal and child mortality in India: A nationally representative mortality survey. Lancet.

[11] Wardlaw T., Salama P., Brocklehurst C., Chopra M., Mason E. (2010). Diarrhoea: Why children are still dying and what can be done. Lancet.

[12] UNICEF, WHO Diarrhoea: Why Children Are Still Dying and What Can Be Done. https://www.who.int/publications/i/item/9789241598415.

[13] Ministry of Health and Family Welfare Health Ministry launches Intensified Diarrhoea Control Fortnight (IDCF)-Intensified Efforts to Reduce Child Deaths Due to Diarrhoea. https://pib.gov.in/newsite/printrelease.aspx?relid=165631.

[14] Shirole T. (2015). Indian Government Launches a Program to Reduce Diarrhea Deaths in the Country. Medindia, Indian Heal. News. https://www.medindia.net/news/indian-government-launches-a-program-to-reduce-diarrhea-deaths-in-the-country-151448-1.htm.

[15] Kapil U. (2002). Integrated Child Development Services (ICDS) scheme: A program for holistic development of children in India. Indian J. Pediatr..

[16] Mihrete T.S., Alemie G.A., Teferra A.S. (2014). Determinants of childhood diarrhea among underfive children in Benishangul Gumuz regional state, north West Ethiopia. BMC Pediatr..

[17] Mohammed S., Tamiru D. (2014). The Burden of Diarrheal Diseases among Children under Five Years of Age in Arba Minch District, Southern Ethiopia, and Associated Risk Factors: A Cross-Sectional Study. Int. Sch. Res. Not..

[18] Lakshminarayanan S., Jayalakshmy R. (2015). Diarrheal diseases among children in India: Current scenario and future perspectives. J. Nat. Sci. Biol. Med..

[19] Azage M., Kumie A., Worku A., Bagtzoglou A.C. (2016). Childhood diarrhea in high and low hotspot districts of Amhara Region, northwest Ethiopia: A multilevel modeling. J. Health. Popul. Nutr..

[20] Sarker A.R., Sultana M., Mahumud R.A., Sheikh N., Van Der Meer R., Morton A. (2016). Prevalence and Health Care–Seeking Behavior for Childhood Diarrheal Disease in Bangladesh. Glob. Pediatr. Health.

[21] Anteneh Z.A., Andargie K., Tarekegn M. (2017). Prevalence and determinants of acute diarrhea among children younger than five years old in Jabithennan District, Northwest Ethiopia, 2014. BMC Public Health.

[22] Asfaha K.F., Tesfamichael F.A., Fisseha G.K., Misgina K.H., Weldu M.G., Welehaweria N.B., Gebregiorgis Y.S. (2018). Determinants of childhood diarrhea in Medebay Zana District, Northwest Tigray, Ethiopia: A community based unmatched case-control study. BMC Pediatr..

[23] Getachew A., Guadu T., Tadie A., Gizaw Z., Gebrehiwot M., Cherkos D.H., Menberu M.A., Gebrecherkos T. (2018). Diarrhea Prevalence and Sociodemographic Factors among Under-Five Children in Rural Areas of North Gondar Zone, Northwest Ethiopia. Int. J. Pediatr..

[24] Zedie F., Kassa D. (2018). Socio-economic, behavioral and environmental factors associated with diarrhea among under five children in health development and non-health development army member mothers in Wondogenet, south Ethiopia. Health Educ. Care.

[25] Melese B., Paulos W., Astawesegn F.H., Gelgelu T.B. (2019). Prevalence of diarrheal diseases and associated factors among under-five children in Dale District, Sidama zone, Southern Ethiopia: A cross-sectional study. BMC Public Health.

[26] Workie G.Y., Akalu T.Y., Baraki A.G. (2019). Environmental factors affecting childhood diarrheal disease among under-five children in Jamma district, South Wello zone, Northeast Ethiopia. BMC Infect. Dis..

[27] Vijayan B., Ramanathan M. (2020). Prevalence and clustering of diarrhoea within households in India: Some evidence from NFHS-4, 2015–2016. J. Biosoc. Sci..

[28] Abuzerr S., Nasseri S., Yunesian M., Hadi M., Zinszer K., Mahvi A.H., Nabizadeh R., Mustafa A.A., Mohammed S.H. (2020). Water, sanitation, and hygiene risk factors of acute diarrhea among children under five years in the Gaza Strip. J. Water Sanit. Hyg. Dev..

[29] Godana W., Mengiste B. (2013). Environmental factors associated with acute diarrhea among children under five years of age in derashe district, Southern Ethiopia. Sci. J. Public Health.

[30] Adane M., Mengistie B., Kloos H., Medhin G., Mulat W. (2017). Sanitation facilities, hygienic conditions, and prevalence of acute diarrhea among under-five children in slums of Addis Ababa, Ethiopia: Baseline Survey of a longitudinal study. PLoS ONE.

[31] Nsabimana J., Mureithi C., Habtu M. (2017). Factors Contributing to Diarrheal Diseases among Children Less than Five Years in Nyarugenge District, Rwanda. J. Trop. Dis..

[32] Mallick R., Mandal S., Chouhan P. (2020). Impact of sanitation and clean drinking water on the prevalence of diarrhea among the under-five children in India. Child. Youth Serv. Rev..

[33] Paul P. (2020). Socio-demographic and environmental factors associated with diarrhoeal disease among children under five in India. BMC Public Health.

[34] Kamath A., Shetty K., Unnikrishnan B., Kaushik S., Rai S.N. (2018). Prevalence, patterns, and predictors of diarrhea: A spatial-temporal comprehensive evaluation in India. BMC Public Health.

[35] Avachat S.S., Phalke V.D., Phalke D.B., Aarif S.M., Kalakoti P. (2011). A cross-sectional study of socio-demographic determinants of recurrent diarrhoea among children under five of rural area of Western Maharashtra, India. Australas. Med. J..

[36] Ahmed S.F., Farheen A., Muzaffar A., Attoo G.M. (2008). Prevalence of diarrhoeal disease, its seasonal and age variation in under-fives in Kashmir, India. Int. J. Health Sci..

[37] Stanly A.M., Sathiyasekaran B.W., Palani G. (2009). A population based study of acute diarrhoea among children under 5 years in a rural community in South India. Sri Ramachandra J. Med..

[38] Gupta A., Sarker G., Rout A.J., Mondal T., Pal R. (2015). Risk correlates of diarrhea in children under 5 years of age in slums of Bankura, West Bengal. J. Glob. Infect. Dis..

[39] Ahmed F., Shahid M., Cao Y., Qureshi M.G., Zia S., Fatima S., Guo J. (2021). A Qualitative Exploration in Causes of Water Insecurity Experiences, and Gender and Nutritional Consequences in South-Punjab, Pakistan. Int. J. Environ. Res. Public Health.

[40] Shahid M., Cao Y., Shahzad M., Saheed R., Rauf U., Qureshi M.G., Hasnat A., Bibi A., Ahmed F. (2022). Socio-Economic and Environmental Determinants of Malnutrition in under Three Children: Evidence from PDHS-2018. Children.

